# A pilot randomized controlled trial of dialectical behavior therapy (DBT) for reducing craving and achieving cessation in patients with marijuana use disorder: feasibility, acceptability, and appropriateness

**DOI:** 10.47626/2237-6089-2020-0123

**Published:** 2021-12-10

**Authors:** Mohammadreza Davoudi, Zahra Allame, Aliakbar Foroughi, Amir Abbas Taheri

**Affiliations:** 1 Department of Clinical Psychology University of Social Welfare and Rehabilitation Sciences Tehran Iran Department of Clinical Psychology , University of Social Welfare and Rehabilitation Sciences , Tehran , Iran .; 2 Department of Clinical Psychology Kermanshah University of Medical Sciences Kermanshah Iran Department of Clinical Psychology , Kermanshah University of Medical Sciences , Kermanshah , Iran .

**Keywords:** Dialectical behavior therapy, marijuana use, feasibility studies, craving, lapse

## Abstract

**Objective:**

To conduct a pilot RCT investigating the feasibility, acceptability, and preliminary efficacy of dialectical behavioral therapy (DBT) for marijuana cessation and craving reduction.

**Methods:**

Sixty-one patients with marijuana use disorder diagnoses were randomly assigned to a DBT group or a control group (psycho-education). Patients completed measures at pre-intervention, post-intervention, and at two-month follow-up. The Marijuana Craving Questionnaire (MCQ) and marijuana urine test kits were used to assess craving and abstinence respectively.

**Results:**

The feasibility of DBT was significantly higher than control group feasibility. In the DBT 29/30 participants completed all sessions (96% retention) and 24/31 control group participants completed all sessions (77% retention) (χ2 = 4.95, p = 0.02). Moreover, 29/30 (96%) participants in the DBT group completed the two-month follow-up and 20/31 (64.5%) control group members completed the two-month follow-up (χ2 = 9.97, p = 0.002). The results showed that patients in the DBT group had significantly higher intervention acceptability rates (16.57 vs. 9.6) than those in the control group. This pattern was repeated for appropriateness rates (p < 0.05). The overall results for craving showed that there was no significant difference between the groups (F = 3.52, p > 0.05), although DBT showed a significant reduction in the “emotionality” subscale compared to the control group (F = 19.94, p < 0.05). To analyze cessation rates, DBT was compared to the control group at the posttest (46% vs. 16%) and follow-up (40% vs. 9.5%) and the results confirmed higher effectiveness in the DBT group for cessation (p < 0.05). Furthermore, among those who had lapsed, participants in the DBT group had fewer consumption days than those in the control group (p < 0.05).

**Conclusions:**

DBT showed feasibility, acceptability, and promising efficacy in terms of the marijuana cessation rate.

**Clinical trial registration:**

Thailand Registry of Clinical Trials, TCTR20200319007.

## Introduction

Marijuana is the most prevalent substance among those reported to be a significant problem among people seeking treatment for substance abuse. ^1^ According to WHO reports, more than 140 million people consume marijuana every year. ^2^ With regard to Iran, recent evidence shows that more than 5% of people consume marijuana every year, predominantly young males. However, in view of the harsh marijuana prohibition policy of the Iranian government, most clinicians estimate that these rates have been hugely underestimated. ^3^ Marijuana, as an illegal drug, is associated with significant physical, psychological, and social consequences. ^4^ Studies have shown that regular and heavy marijuana use patterns correlate with increased risk of mood disorders, anxiety, and psychotic episodes and although causality has not been demonstrated, these patterns can increase the course of mental health problems. ^5^ Also, several medical problems such as respiratory system deficits, stroke, myocardial infarction, and digestive tract cancers are associated with marijuana use patterns, especially among those with marijuana use disorder (MUD). ^6 , 7^ Approximately one in three marijuana users meet the criteria for MUD based on the DSM-5, and this proportion is rising. ^8^ One of the most important psychological problems in substance use disorder treatment is craving. Craving is a factor identified as the root cause of relapses and treatment failures. ^9 , 10^ MUD patients report visual, tactile, and olfactory cues related to craving and compulsivity sensations. ^11^ Based on these results, clinicians have tried to treat patients with marijuana use disorder.

To date, the Food and Drug Administration (FDA) in the United States has not approved any psycho-pharmacotherapy for MUD, and therefore psycho-social interventions have received particular attention. ^12^ The most widely used psychological treatment in the substance use disorder (SUD) context is cognitive-behavioral therapy (CBT). ^13^ Results showed that CBT is somewhat effective for SUD, but that most patients with MUD do not achieve cessation and are not motivated to continue skills training during follow-up. Relapse rates therefore remain a considerable limitation of treatment. ^10 , 13^ One of the main reasons for this low success rate lies in the limitations of CBT. First, CBT protocols do not focus on comorbid problems, whereas most patients with SUD have at least one psychiatric or psychological problem. Secondly, cognitive restructuring may not be useful for all SUD patients. Some patients may be unable to restructure their dysfunctional cognition and core beliefs despite receiving CBT. ^10 , 12 , 13^ Furthermore, emotion regulation deficits are strongly associated with increased addictive behaviors such as SUD. With emotion regulation, people adjust their emotional experiences related to distressing and unpleasant events. Emotion regulation is essential for successful coping with environmental demands and personal welfare. ^14^ On the behavioral level, studies have found that marijuana craving cues are strongly associated with deficits in regulation of negative affect and emotions. ^14 , 15^ Also, on the neural level, during reappraisal of negative stimuli, patients with MUD and regular users have shown altered neural activity and functional connectivity. Moreover, marijuana use is related to dysfunctions in the amygdala and in amygdala-dorsolateral prefrontal cortex (DLPFC) coupling activity. ^15^ Together, these findings demonstrate that emotion-based psychotherapy must manage comorbid problems and eliminate the limitations of CBT.

One of the psychotherapies in the third wave behavioral therapy cluster is dialectical behavior therapy (DBT). DBT has been described as intervention in emotion regulation deficits by focusing on dangerous impulses in borderline personality disorder and substance use disorder. The goals of DBT include improving and regulating emotions as a primary mechanism of change. DBT is a trans-diagnostic treatment and suitable for comorbid problems. DBT trains skills including distress tolerance, interpersonal effectiveness, emotion regulation, and mindfulness. Overall, in the context of SUD, DBT teaches emotion regulation skills to decrease engagement in pathological emotion regulation strategies. It also intervenes in low quality of life situations, reduces drug-seeking behavior, and helps patients function adaptively by accepting unpleasant emotions such as craving. ^9 , 10 , 14^

Research literature shows the efficacy and effectiveness of DBT in various comorbid problems and diseases such as suicide, ^16^ forensic psychiatric patients, ^17^ and irritable bowel syndrome. ^18^ Nevertheless, studies have reported contradictory results for the effectiveness of implementing DBT in various SUD populations. ^19 , 20^ Furthermore, the literature has recommended using larger samples, clearer instruments to measure outcome variables, and specific and integrated protocols. ^21^

Additionally, according to our investigations, no DBT randomized clinical trials have been conducted that investigated cessation in MUD patients (with or without comorbid problems). A DBT intervention aimed at increasing the cessation rate and reducing craving among MUD patients was developed for this study.

This pilot trial investigated the feasibility and preliminary efficacy of DBT relative to a psycho-education intervention that was controlled for time duration and attention. Feasibility was assessed via satisfaction and session completion rates. Preliminary efficacy was evaluated via the impact of DBT on cessation rate and reduction of consumption rates, compared to the psycho-education intervention. Although craving and acceptance of craving are not the primary goals of DBT, they were also compared across the two interventions.

## Methods

### Trial design

This study was designed as a controlled randomized clinical trial, including pretest, post-test, and two-month follow-up phases.

### Sample size

Since the sampling method comprises snowball sampling and strict eligibility criteria were applied, on the basis of data from similar studies ^10^ it was determined that at least 20 participants were needed in each group. However, in view of the predicted retention rates, we selected 30 patients for each group.

### Selection criteria

The inclusion criteria were as follows: 1) diagnosis of marijuana use disorder; 2) age 18 years or over; 3) no current or past history of major psychiatric disorders; 4) no other concurrent SUD treatment; and 5) willingness to attend intervention sessions, complete surveys, and take tests (questionnaires and urine test kits).

Exclusion criteria were as follows: 1) unwillingness to participate; 2) not participating in intervention sessions for more than two weeks; 3) starting secondary psychotherapy; and 4) consuming methamphetamine, amphetamine, cannabis, methadone, benzodiazepines, or morphine during the research stages.

### Participants, procedures, and randomization

Since there are no cannabis use disorder treatment centers in Iran, there is no specific place to select patients. Furthermore, patients at drug treatment centers are referred for treatment of other substance use disorders and comorbidity of drug use is one of the exclusion criteria for this study, since it could lead to misleading results. Therefore, the relatives and acquaintances of those who had been referred to the drug treatment center were interviewed. From November 1, 2019, to November 5, 2019, 15 relatives and family members of drug users referred to drug treatment centers were diagnosed with MUD at this stage. Then, using snowball sampling, after 15 days of investigation, a total of 83 patients were diagnosed with MUD. Seventy-five of the 83 MUD patients who were approached consented, eight declined to participate, and 14 were ineligible. The primary reasons for declining were anxiety about addiction stigma and time constraints. Most of the ineligible patients had multiple illicit use disorders, so they did not meet the study criteria. Therefore, 61 patients completed the baseline assessment and were included in the current analyses. These patients were randomly assigned to each group using a random number table. The interventions were implemented from December 1, 2019, to March 20, 2020. The follow-up phase started on March 21, 2020, and ended on May 20, 2020, (at two months’ follow-up). In order to test for exclusion criteria before each session, a six-drug test kit for methamphetamine, amphetamine, cannabis, methadone, benzodiazepines, and morphine was administered to individuals using urine samples.

### Blinding

Both groups were blind to the existence of another group in the study. However, patients were informed about participating in research but not about another group. One day after the end of treatment, the post-test was carried out by mental health technicians with a master’s degree in psychology.

### Outcome measures

#### Abstinence

A marijuana urine test kit prepared by Kian Teb Company (officially licensed by the National Medical Device Directorate IR. IRAN) was used to identify abstainers.

#### Marijuana smoking

A self-report scale was designed for patients who had lapsed during the post-test follow-up. On this scale, patients indicate the number of days of consumption over 30-day periods. The first thirty days after the last intervention session was considered the post-test smoking period and the second-month follow-up was considered as the follow-up marijuana use period.

#### Craving

The Marijuana Craving Questionnaire (MCQ) short-form is a 12-item self-report questionnaire with ten items for subjective assessment of cannabis craving. The scale covers 4 factors: compulsivity, emotionality, expectancy, and purposefulness. According to how patients were thinking or feeling ‘’right now,’’ they placed checkmarks on the questionnaire to endorse responses ranging from 1 or strongly disagree to 7 or strongly agree. Results showed that this questionnaire’s internal consistency is adequate (α = 0.90). The measure was administered following a 12-hour deprivation period. The typical onset of marijuana craving and withdrawal symptoms is observed within approximately one day of cessation and so the current paper’s questionnaire scores can be conceptualized as an index of the propensity to experience marijuana craving following deprivation. ^22^ In Iranian MUD patients, the MCQ had internal consistency of α = 0.87. Details of the MCQ’s psychometrics properties will be published as a separate study as soon as possible.

#### Acceptability

The Acceptability of Intervention Measure (AIM) was employed to measure the acceptability of interventions. AIM response items are measured on a 5-point Likert scale (from Completely Disagree with 1 point to Completely Agree with 5 points). The mean of points scored for each item is taken as the final score. This questionnaire developed by Weiner et al., and they reported Cronbach’s ɑ = 85 for internal consistency. ^23^

#### Appropriateness

The Intervention Appropriateness Measure (IAM) was used for Appropriateness. The IAM consist of a four-item scale that measures perceived intervention appropriateness. Items are measured on a five-point Likert scale (Completely Disagree to Completely Agree), and the mean of points scored for each item is taken as the final score. Higher scores mean that the participant feels this intervention is more appropriate for him/her. For this tool, Cronbach’s ɑ = 0.91 and all Factor Loadings are reported as higher than 0.8. ^23^

## Intervention

### Dialectical behavior therapy

DBT is a group intervention consisting of 16 sessions (meeting once a week for 90 minutes) with one psychotherapist and her co-therapist. The intervention protocol is an adaptation of DBT for SUD based on three basic manuals. ^10 , 24 , 25^ The primary objective of the DBT is to reduce dysfunction in emotion regulation and craving via increasing cessation rates and improving skills. A psychotherapist with a PhD delivered the intervention sessions (with a psychologist as co-therapist) and they were blind both to the existence of another group and to the study objectives. Table 1 shows the content covered in each DBT session.

**Table 1:** DBT content per session

Cessation	Content
Pre-session	Explanation of dialectical behavioral therapy, principles, and goals. Brief introduction to the content of each session. Familiarity with participants. Participants are given an intervention booklet to read at home.
1st session (mindfulness 1)	Introduce the concept of mindfulness and three mental states (wise, reasonable, emotional) and their relations with substance use.
2nd session (mindfulness 2)	Teach two clusters of mindfulness skills. The first includes viewing, participation, and description. The second includes a non-judgmental stance and inclusive self-consciousness.
3rd session	Summarize the mindfulness sessions – definition of addiction, standard therapies of addiction, introduction to and teaching of dialectical avoidance technique. Review the positive and negative aspects of abstinence. Explanation and investigation of relapse and its causes. Explaining the skill of the pure mind, the addicted mind, the types of behaviors related to the pure mentality and the addicted mentality, and preparing a list of supporters.
4th-5th sessions (Distress tolerance)	Teaching distraction strategies with five skills include activities, comparisons, emotions, thoughts, and enjoyment. Through enjoyable activities, focusing on work or other topics, counting, leaving the situation, paying attention to daily tasks, distracting from thoughts, and self-harm behaviors – teaching and training self-soothing with five senses.
6th-7th sessions (Emotion regulation)	Definition of emotion, how emotions work, familiarity with emotion regulation skills. Emotion Identification Exercise, Emotion Registration Exercise. Identifying barriers to experiencing emotion in a healthy way and ways to overcome these barriers. Teaching creating short-term positive emotional experiences for experiencing positive emotional states.
8th-10th sessions (Emotion regulation and distress tolerance in an MUD context)	Explain craving and its connection to the experience of emotions. Introducing methods for identifying values. Importance of committed action based on a list of essential values in life. Develop new coping strategies in response to unpleasant emotions, sensations, and cognitions, especially craving as a multidimensional problem and teaching problem solving and behavior analysis.
11th session	Basic acceptance technique training. Introduce living in the present moment techniques.
12th-13th sessions	Interpersonal effectiveness training. Participants learn assertiveness skills about substance users. Other skills include non-verbal communication, verbal communication, and problem-solving, decision-making, and listening skills.
14th-16th sessions	Review of sessions. Elimination of ambiguities. Exercising skills in the presence of other people.

### Psychoeducation

This option is more ethical than not offering any intervention to the control group. A psychiatrist with five years of experience in addiction psychotherapy implemented this intervention. This intervention includes problem-solving skills, assertiveness, and craving management in eight sessions. Thepsychoeducation intervention was used to provide a basis for comparison with only those elements of the DBT intervention that are different from other psychotherapies. This intervention is utilized for MUD and health-related problems. The intention of this intervention is to provide individuals struggling with cravings and substance use disorder the knowledge needed to comprehensively appreciate their problems and the empowerment needed to cope with them. The psychoeducation intervention included information about the dangers of marijuana and the therapists also provided a pamphlet containing techniques for reduction of craving. ^14 , 26 , 27^

### Therapists and treatment adherence

To enable adherence to the principles of DBT to be checked, audios of the sessions were recorded with the consent of all participants. A DBT researcher and psychotherapist who was not involved in the treatment groups checked session content afterwards. Sessions were divided into 15-minute modules that were chosen for adherence checks at random. Treatment stance and occurrence and depth of DBT processes were appraised. Based on the treatment manual, modules were rated for adherence level as either adequate or not adequate. The majority (83%) were judged to have been conducted adequately.

### Statistical method

Demographic information was gathered and reported as frequencies, means, and standard deviations and repeated measures ANOVA and chi-square tests were conducted for the outcomes using SPSS software, version 26.

## Ethical considerations

Written informed consent was obtained from all participants before initiation of the research. The tools used in this study were all filled-out anonymously, and an ID code was used to maintain the confidentiality of personal information (Ir.kums.rce.1398.1203). At the end of the research process, dialectical behavior therapy was also provided to the control group. This study is registered with the Thailand Registry of Clinical Trials (TCTR20200319007).

## Results

### Feasibility

**Figure 1:**
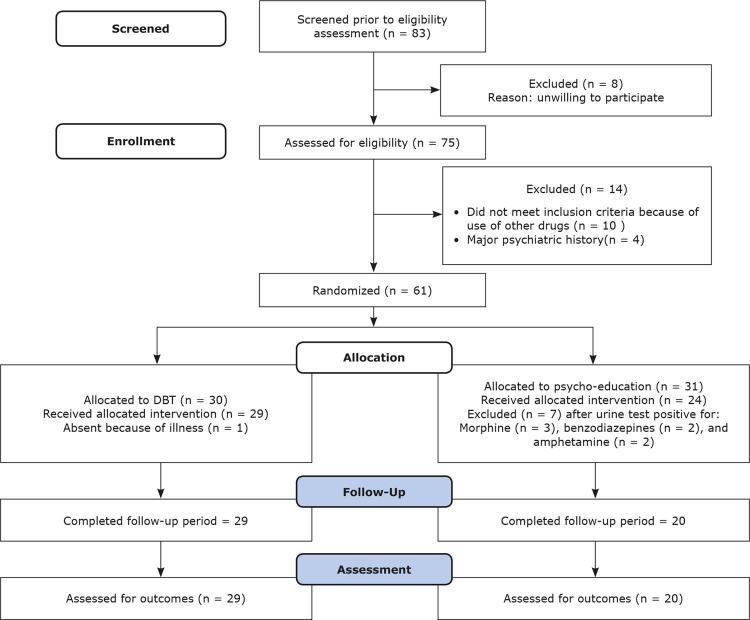
Consort diagram.

In the psycho-education group, 24/31 participants completed all sessions, compared to 29/30 members of the DBT group (retention rates: 77% in the control group vs. 96% in the DBT group). Additionally, 96% (29/30) of the DBT group members completed the two-month follow-up, whereas 64.5% (20/31) of the control group completed follow-up ( Figure 1 ). The chi-square test was applied, showing associations between group and retention, with χ2= 4.95, p = 0.02 for post-treatment and, χ2= 9.97, p = 0.002 for the follow-up phase. Consequently, DBT retention rates were significantly higher than psycho-education retention rates at post-treatment and follow-up.

### Acceptability and appropriateness

To enable assessment of the acceptability and appropriateness of intervention, patients completed the AIM and IAM scales in the post-treatment phase. The acceptability scores were 16.57 for DBT and 9.6 for the control group (p < 0.05). The appropriateness scores were17.03 for DBT and 10.7 for the psychoeducation group (p < 0.05). Since there are no standards for these measures, these points were transferred to Likert-based questionnaire scales. For acceptability, the results equated to ‘’agree’’ for the DBT group versus ‘’neither agree nor disagree’’ for the psychoeducation group. For appropriateness, the results equated to ‘’completely agree’’ for the DBT group versus ‘’neither agree nor disagree’’ for the psychoeducation group.

### Participant characteristics

Participants’ demographic variables are shown in Table 2 . Analyses showed that there were no significant differences between the two groups regarding these variables. It should be noted that since over 97% of the participants were male from the beginning, the results were reported only for men.

**Table 2:** Mean and standard deviation of demographic variables in the intervention and control groups at the test phase

Variable	Intervention group	Control group	p-value
Educational level*			0.2
No higher education, n (%)	2 (6)	6 (19)	
Diploma, n (%)	14 (46)	15 (48)	
University student or graduate, n (%)	14 (46)	10 (33)	
Age ^†^	25.6 (5.67)	27.19 (7.48)	0.3
Months of marijuana use	19.53 (5.9)	17.48 (6.03)	0.1
Craving (total) ^†^			
Pre-test	45.2 (8.3)	47.9 (10.2)	0.2
Post-test	42.13 (7.7)	44.48 (8.1)	0.1
Follow-up	42.66 (9.25)	45.8 (8.4)	0.1

Data presented as mean (standard deviation), unless otherwise specified.

* Chi-square test.

^†^ Independent *t* test.

### Efficacy outcomes

The hypothesis of equal covariance matrices was examined for craving (Box’s M = 3.63, P = 0.74). The results of this test indicate homogeneity of covariance matrices. Mauchly’s test of sphericity also showed that the sphericity assumption was not violated (p = 0.42 and Mauchly’s W = 0.97).

The results of the intergroup test and intergroup relations are also presented in Table 3 . As shown in Table 3 , the effect levels for craving (F = 3.52, p > 0.05) suggest that there is no significant difference between groups. These results were repeated for three of the subscales of craving: compulsivity, expectancy, and purposefulness. However, the emotionality subscale results showed a significant reduction in the DBT group compared to the control group 10.6 vs. 14.4 in the post-test and 10.43 vs. 13.26 in the follow-up phase (F = 19.94, p < 0.05).

**Table 3:** Repeated measures ANOVA for variables for the DBT group and control group in the pre-test, post-test, and follow-up

Variable/source	Type III sum of squares	df	Mean square	F	Sig	Partial eta squared	Observed power
Craving							
Tests of within-subjects effects							
Factor1	347.696	2	173.84	2.6	0.07*	0.04	0.512
Factor1 × group	4.76	2	2.38	0.036	0.11	0.001	0.055
Error (factor1)	7845.154	118	66.48				
Tests of between-subjects effects							
Group	341.084	1	341.08	3.52	0.06 ^†^	0.056	0.455
Error	5708.79	59	96.75				
Emotionality							
Tests of within-subjects effects							
Factor1	257.33	2	128.65	11.69	0.00 ^†^	0.165	0.9
Factor1 × group	74.70	2	37.35	0.37	0.03*	0.05	0.63
Error (factor1)	1297.81	118	10.99				
Tests of between-subjects effects							
Group	283.89	1	283.89	19.94	0.00 ^†^	0.25	0.9
Error	839.906	59	14.23				

* Significant to 0.05.

^†^ Significant to 0.01.

With regard to cessation, the results indicated that DBT achieved a higher rate of cessation than the control treatment in both the post-test and at follow-up, ( Table 4 ) (p < 0.05). It was also found that among those who continued to use the drug, the number of use days per month in the post-test and the follow-up periods (two months) was significantly lower in the intervention group than the control group.

**Table 4:** Cessation and consumption between groups

	DBT	Control	p-value
Cessation*			
Post-test	14 (46%)	5 (16%)	**0.01** ^‡^
Follow-up	12 (40%)	3 (9.5%)	**0.006** ^‡^
Number of days use ^†^			
Post-test	2.43±1.8	7.5±5.03	**0.00** ^‡^
Follow-up	3.44±1.91	8.75±3.27	**0.00** ^‡^

Bold p-values are significant at critical levels.

* The chi-square test was applied.

^†^ T test for independent samples.

^‡^ Significant to 0.01.

## Discussion and conclusion

This study examined the feasibility, acceptability, and preliminary efficacy of a 16-session DBT intervention to address craving and achieve cessation in MUD patients. This intervention showed strong evidence of feasibility. Moreover, acceptability and appropriateness rates in the DBT group were high and adequate. The results showed that DBT is a promising intervention for marijuana cessation in patients with MUD. Although this study is the first RCT of DBT for MUD, the scientific literature about DBT for other addictive behaviors reports similar results.

Rezaei et al. found that DBT significantly improved craving among methadone users. Their result showed that DBT could reduce methadone usage and improve emotion regulation. ^28^ Moreover, another study showed that implementation of DBT with alcohol-dependent patients improved alcohol-related behavior and emotional deficits, which is similar to the results of the present study. ^29^ However, the results for craving showed there was no significant difference between groups. With regard to this finding, our result differs from the majority of other research findings. For example, Rezaei et al. found that DBT significantly improved craving among methadone users. ^10^ This result was also repeated in Rabinovitz’s paper. ^30^ One of the main reasons for this difference lies in the finding in the present study that DBT had greater improvement in the emotionality subscale of craving (p < 0.5). Since the most important structure of marijuana craving is its emotional dimensions, ^5 , 14^ the lack of changes in other subscales resulted in non-significance for the overall craving scale score. The results of the present study with relation to craving are therefore somewhat co-directional with the findings of previous studies. On the behavioral level, other studies found that Marijuana craving cues were strongly associated with deficits in regulation of negative affect and emotions. ^14 , 15^ Also, when neural levels were assessed during reappraisal of negative stimuli, patients with MUD and regular users showed altered neural activity and functional connectivity. Furthermore, being a marijuana user was related to dysfunctions in the amygdala and in amygdala-DLPFC coupling activity. ^14^ Taken together, these findings demonstrate that emotion regulation problems and craving are prevalent in MUD patients and can interfere with the cessation process. Since DBT is a third-wave behavioral therapy, it has a strong emotional basis. This therapy encompasses three emotion-based goals: understanding emotions, reducing emotional vulnerability, and reducing emotional suffering. Patients are helped to understand that unpleasant emotions are a normal part of life and that accepting their existence is more healthy than trying to avoid controlling them. ^10 , 28^ Overall, DBT is an emotion regulation method that helps patients learn, understand, and label emotions, reducing emotional vulnerability and emotional suffering. These skills help MUD patients to label emotions related to craving. This improvement in emotional states can improve dysfunctions in the amygdala and in amygdala-DLPFC coupling activity. ^9 , 31 , 32^

Along the same lines, it also improves emotional craving-related brain structures and reduces impulsive behavior (e.g., lapses). Also, with the ‘’distress tolerance’’ component of DBT, patients learn to live with destructive emotions to accept unpleasant craving situations. ^32 , 33^ Therefore, by increasing craving, they no longer consume marijuana immediately. ^22 , 24^ Similarly, other DBT components teach MUD patients reinforcement management and problem-solving skills that can help them to reduce marijuana consumption. ^34 , 35^

## Conclusion

To conclude, DBT demonstrated adequate feasibility, acceptability, and appropriateness for patients with marijuana use disorder. Moreover, DBT also exhibited significant efficacy compared to the control group for achieving cessation and reducing emotion-related craving. Even in patients who could not achieve abstinence, DBT led to a reduction in marijuana consumption rates. These findings persisted at two-month follow-up.

### Limitations and future directions

Despite these positive results, the present study also has some limitations. First, in order to evaluate the most significant treatment components (such as mindfulness and distress tolerance), no groups received the third wave versions of other therapies (ACT or MBSR). This study only had a two-month follow-up period and could not conduct long-term evaluation due to the study site’s medical and infrastructure conditions. It is recommended that future research should examine mediating and confounding variables to investigate the results of similar research to the present study. Other factors affecting relapse and recurrence could also be examined. Moreover, women should be investigated so that gender-related implications can be determined.
